# Epidemiology of* Plasmodium* and Helminth Coinfection and Possible Reasons for Heterogeneity

**DOI:** 10.1155/2016/3083568

**Published:** 2016-03-22

**Authors:** Abraham Degarege, Berhanu Erko

**Affiliations:** Aklilu Lemma Institute of Pathobiology, Addis Ababa University, P.O. Box 1176, Addis Ababa, Ethiopia

## Abstract

Understanding the impact of helminth infections on clinical malaria is useful for designing effective malaria control strategies. Plenty of epidemiological studies have been conducted to unravel the nature of interactions between* Plasmodium* and helminth infection. Careful broad summarization of the existing literature suggests that* Schistosoma mansoni* and hookworm infections may increase the risk of clinical malaria and associated morbidities, but* Trichuris trichiura* infection is not associated with the occurrence of clinical malaria and related outcomes. However, findings about effect of* Ascaris lumbricoides* and* Schistosoma haematobium* infection on clinical malaria are contradictory. Furthermore, the nature of relationship of helminth infection with severe malaria has also not been determined with certainty. This review summarizes the findings of epidemiological studies of* Plasmodium* and helminth coinfection, placing greater emphasis on the impact of the coinfection on malaria. Possible reasons for the heterogeneity of the findings on malaria and helminth coinfections are also discussed.

## 1. Introduction

Although the nature of interaction remains uncertain, studies showed that an apparently true biological association exists between* Plasmodium* and helminths when they coexist in a host [1, 2]. Hence, the presence of helminth can affect the risk of malaria and severity of the disease; or the occurrence of* Plasmodium* infection may in turn impact the upcoming helminth infections and related morbidities [1, 2]. As a result, disease due to one of these parasites could be exacerbated or ameliorated due to the cooccurrence of the other species resulting in synergistic or antagonistic impacts on the infected host.

Despite such a bidirectional nature of interactions between the two groups of parasites [1], studies usually evaluate the impact of helminth coinfection on malaria. This could be due to the perceived larger public health impact of malaria compared to helminth infections. In addition to this, helminths are known for their strong immune-modulatory impact on other coinfecting parasites compared to* Plasmodium* [3]. Helminths also reduce the amount of red blood cells necessary for* Plasmodium* to reproduce. Moreover, anaemia caused by helminth infection can lead to cardiovascular compensation, hyperventilation of CO_2_, and increased lactates which can make the host more attractive to mosquitoes [4].

This paper provides a brief review of epidemiological studies on helminths and malaria interactions with emphasis on the impacts of the coinfection on malaria. Possible reasons for the inconsistency of the findings on malaria and helminth coinfections are discussed. Scholar search in PubMed, Embase, and Google was performed without restricting to language, publication date, study design, and nature of the study participants (e.g., age, sex, pregnant, and health status). Combinations of key words such as “helminths and malaria or* Plasmodium*”, “*Ascaris* and malaria or* Plasmodium*”, “*Schistosoma* and malaria or* Plasmodium*”, “hookworm and malaria or* Plasmodium*”, “*Trichuris* and malaria or* Plasmodium*”, and “*Strongyloides* and malaria or* Plasmodium*” were used to search relevant references. Studies on* Plasmodium* and helminth coinfections in animals were excluded. In addition, human studies on the immunological evidences of* Plasmodium* and helminth,* Ascaris*,* Schistosoma*, hookworm, or* Trichuris* interactions were excluded. More than 7560 published articles were identified following the literature search. After exclusion of duplicates and screening of the titles and abstracts, a total of 107 articles were found that fulfill the inclusion criteria. About 50 articles were further excluded after reading the full-text. The characteristics of the studies on epidemiology of* Plasmodium* and helminth coinfection are provided in Table 1 in Supplementary Material available online at http://dx.doi.org/10.1155/2016/3083568.

## 2. *Schistosoma* and Malaria

Many studies have been conducted to evaluate the nature of the interactions between* Schistosoma* and* Plasmodium*. More than 20 of these studies were conducted under natural conditions in humans. Of these studies, five looked at immunology, thus excluded from the current epidemiology focused review.

Most of the epidemiological studies were conducted among children infected with* P. falciparum*. However, findings about malaria prevalence or incidence, density of the parasite, and associated morbidities during* S*.* haematobium* infection were heterogeneous. While some studies reported decreased malaria prevalence [5], incidence [6],* Plasmodium* density [5], disease severity [5], and associated splenomegaly [7], others reported increased prevalence or risk of* Plasmodium* infection and density of the parasite [7–10] or low haemoglobin level [11] and enlarged spleen [12] among individuals infected with* S*.* haematobium*. Still, other studies reported similar level of prevalence of* Plasmodium* infection [13, 14] or density of the parasite [6, 15, 16] and haemoglobin level [6] among children infected and uninfected with* S. haematobium*.

On the other hand, prevalence [17, 18] or incidence [19, 20] and density of asexual [21] or gametocyte [22] stages of* P*.* falciparum* infection and related anaemia [20] or hepatosplenomegaly [23] increased in individuals infected with* S*.* mansoni* alone [17, 19, 21, 23] or both* S*.* mansoni* and* S. haematobium* [20, 22]. However, two studies reported lack of association between prevalence of falciparum malaria and* S*.* mansoni* infection [13, 24]. The nature of association between* Schistosoma* and malaria seems to vary with the age of the individuals [6] and intensity of* Schistosoma* [19, 25] infection.

## 3. *Ascaris lumbricoides* and Malaria

Randomized controlled trials in Madagascar and Comoros Islands reported increased* P*.* falciparum* incidence and density of the parasite after treatment of children for* A*.* lumbricoides* infection [26–28]. Similarly, a cohort study in Brazil documented a lower drop in haemoglobin level among children coinfected with* P*.* vivax* and* A*.* lumbricoides* compared to children infected with only* P. vivax* [29]. In addition, cross-sectional studies in pregnant women, children, and adults reported association of* A. lumbricoides* infection with a low prevalence [30] or incidence [31] and density of* Plasmodium* infection [32].* A. lumbricoides* infection was also negatively correlated with the occurrence of cerebral malaria and body temperature among patients in Thailand [33, 34].

On the other hand, in a longitudinal study among pregnant women in Gabon and a case control study in Thailand, patients reported association of* A. lumbricoides* infection with increased incidence of malaria [35]. In addition, positive association between* A. lumbricoides* infection and prevalence of malaria was observed among patients in Ethiopia and pregnant women in Ghana [17, 36]. Severe malaria was also found to be more common among children infected than uninfected with* A. lumbricoides* [37]. On the other hand, other studies documented lack of association between* A. lumbricoides* infection and prevalence [13, 38–41] or incidence [42] of malaria and* Plasmodium* density [29]. Overall, findings about the effect of* A. lumbricoides* infection on clinical or severe malaria are inconsistent and it is difficult to make a clear conclusion about the nature of relation between the occurrence of* A. lumbricoides* infection and the risk of malaria based on the existing evidences.

## 4. Hookworm and Malaria

Hookworm is widely distributed in most tropical regions where malaria is endemic [43]. As a result, malaria and hookworm coinfection is common in many parts of the world especially in tropics and subtropics [44]. Moreover, hookworm is a known cause of anaemia and could strongly predict* Plasmodium* infection and associated morbidities [4].

Cross-sectional studies among pregnant women in Thailand, Ghana, and Uganda reported increased malaria prevalence during hookworm coinfection [30, 36, 45]. Hookworm infection was also associated with increased malaria prevalence [13, 41, 46, 47] and* Plasmodium* density [22, 32] among children in Zimbabwe, Ethiopia, Uganda, Kenya, Côte d'Ivoire, and Colombia. On the other hand, some studies showed lack of association between hookworm infection and prevalence [24, 31, 48] or incidence [42] of malaria and* Plasmodium* density [29]. Yet, the existing epidemiological evidences tend to suggest positive association between hookworm infection and occurrence of clinical malaria and associated morbidities.

## 5. *Trichuris trichiura* and Malaria

Unlike other helminth species, there are few epidemiological studies examining the relationship between* T. trichiura* infection and malaria. Perhaps this could be due to the restricted distribution of* T. trichiura* infections in the equatorial regions of Africa which may have resulted in a decreased risk of coinfection with* Plasmodium* [44]. Majority of the studies on* T. trichiura* and* Plasmodium* coinfection showed lack of association between the two groups of parasites. Whilst two studies observed association of* T. trichiura* infection with high prevalence of* P. falciparum* infection among patients in Thailand and Ethiopia [17, 49], three studies among pregnant women in Ghana, Kenya, and Uganda [36, 39, 45], one study among patients in Columbia [41], and another study among primary school children in Zimbabwe [13] did not show relationship between* T. trichiura* infection and the occurrence of malaria or related outcomes. Other studies also reported lack of association between* T. trichiura* infection and malaria incidence [42] or* Plasmodium* density [29, 32] among school-age children and adults. This suggests that* T. trichiura* infection may not affect the occurrence of clinical malaria or associated morbidities. The two studies which showed positive association between* T. trichiura* infection and malaria were conducted among individuals visiting the outpatient clinics. Patients may have other infections or health problems that might have confounded/distorted the direction of relationship between these two groups of parasites.

## 6. Pooled Intestinal Helminth and Malaria Data

Some studies considered pooled data of different helminth species as one variable while evaluating the nature of interactions between* Plasmodium* and helminths rather than considering intestinal helminth species independently. Although variation may exist in their pathogenicity, different helminth species have similar immunopathological impact on hosts. Most gastrointestinal helminths and* Plasmodium* affect host nutrition in a similar manner. Hence, it seems plausible to consider different gastrointestinal helminth species together while assessing the impact of helminth coinfection on malaria.

While some studies reported increased prevalence [17, 36, 50–52] or incidence [53–55] of malaria and gametocyte carriage [55], some studies reported lower occurrence of clinical and severe malaria [32, 56–58] in helminth-infected individuals compared to the uninfected ones. Two studies also reported decreased reticulocyte count and haemoglobin concentration among individuals infected with helminths and* Plasmodium* [59, 60]. Still, other studies reported lack of association between intestinal helminth infection and prevalence [30, 38, 61] or incidence [29, 42, 62] and density of* Plasmodium* infection [15].

## 7. Factors Contributing to the Heterogeneity of the Findings about Malaria and Helminths Coinfection

Factors related to methodology, environment, host, and parasite may explain the lack of uniformity of the findings about the nature of relationship between helminth and malaria.

### 7.1. Methodological Factors

Most previous studies on malaria and helminth coinfection were not uniform in terms of design and methods used for assessing* Plasmodium* and helminth infection, sample size, and the degree to which they control the effect of confounders. They were either cross-sectional, case control, or retrospective analysis of data collected previously for other purposes. Hence, it is difficult to confirm whether helminth infection occurred before the occurrence of malaria. Additionally, sample sizes in some previous studies were small. This might have reduced the power of the studies to detect differences in the occurrence of malaria and associated morbidities between helminth-infected and uninfected individuals. Variation also existed among previous studies on how the study subjects were selected and diagnosed for parasitic infection. Single Kato-Katz technique is less sensitive for examining light intensity helminth infection [63]. In addition, the performance of microscopy in the diagnosis of* Plasmodium* infections relies on blood film quality and experience of the examiner [64]. Thus, intensity of infection could be underestimated or light infections could be missed and* Plasmodium* species may not be correctly identified when the examiner is less experienced.

While intensity and species of helminths are central determinants of infection-related impact on clinical and immune functions of malaria [1, 2], studies usually fail to consider these factors while testing the relationship between the two groups of parasites. Furthermore, some studies did not control for the effect of socioeconomic conditions, place of residence, housing condition, education status, and nutrition status while examining the nature of relationship between the two groups of parasites. Thus, results obtained could have been distorted.

The nature of relationship between helminth and malaria could vary based on the type of helminth species [1, 2]. Hence, analyzing data after combining different helminth species into a single group may yield a different result. If two or more helminth species which can associate with malaria positively and negatively coexist, one may cancel or suppress the effect of the other. For example, in a study by Boel et al. [30], stratified analysis based on the type of helminth species showed that incidence of malaria is positively associated with hookworm infection but negatively associated with* A. lumbricoides* infection. However, this relationship was not maintained when data was analyzed after pooling the different helminth species in one group. Another study also showed negative correlation between* S. haematobium* and* Plasmodium* density, but this relationship was not maintained when analysis was done after pooling different helminth species together [15].

### 7.2. Environmental Factors

Contradictions in findings about the nature of relationship between malaria and helminth could also be due to variation among different geographic locations in their degree of endemicity for malaria and helminth. In areas where there is frequent exposure to malaria, strong immunity will be developed that can be affected during helminth coinfection. In contrast, malaria immunity will be less developed; therefore, the effect of helminth coinfection will be minimal when transmission intensity is low. Indeed, a study in Uganda, where malaria transmission is low, failed to show any association between helminth and malaria even when data were analyzed after stratifying by the type of helminth species and intensity of infection [42].

### 7.3. Host Factors

In addition to methodological and environmental variations, previous studies vary in the nature of the study population. While most studies were conducted in children, some studies were conducted in adults. Some studies also involved pregnant women or immunocompromised individuals who were malnourished or had other viral, bacterial, parasitic, or chronic infections. Moreover, the genetic makeup of individuals, which may affect susceptibility of individuals to malaria, was likely different among the study population in previous studies. These factors may confound the nature of relationship between these two groups of parasites.

### 7.4. Parasite Factors

Intensity and species of helminths and* Plasmodium* were not similar among studies examining malaria and helminth interactions. Although different helminth species affect the immune system in similar manner, variation may exist in their degree of potency [3]. For example,* S. mansoni* is known for its strong influence on the immune system [3]. In addition, some helminth species such as hookworm destroy RBCs to a level where* Plasmodium* cannot replicate [44]. Intensity of helminth infection is also important in the nature of association [1].

Similarly, the types of* Plasmodium* species could also be indispensable in evaluating the nature of relationships between helminths and malaria.* P. falciparum* and* P. vivax* vary in their pre-erythrocyte immunity profile [65], suggesting differences in their degree of association with helminths. Indeed, a study was able to confirm association between different intestinal helminth species and* P. falciparum* malaria but failed to confirm the association among individuals infected with* P. vivax* [17].

## 8. Conclusions and Recommendations

Studies have shown that helminth infection may affect the epidemiology of malaria. Careful broad summarization of the existing evidence suggests that* S. mansoni* and hookworm infections may increase the risk of clinical malaria and associated morbidities, but* T. trichiura* infection is neither associated with the occurrence of clinical malaria nor associated with the morbidities. However, findings about association of* A. lumbricoides* and* S. haematobium* infection with clinical malaria are contradictory. Findings about the nature of relationship of helminth infection with severe malaria are also heterogeneous. It is indicated that most previous studies had limitations in methodology and design and there is a possibility that different socioeconomic and environmental conditions could confound the nature of interaction between helminth and malaria. Thus, well designed randomized controlled clinical trials involving periodic treatment with anthelmintic treatment from well-characterized populations are indispensable to make firm conclusion about the effect of helminth infection upon clinical or severe malaria and related morbidities. This will help to design effective disease management program. To make the conclusion more robust, studies should also focus on immunological analysis of the interaction considering different helminth species independently. Additionally, future studies should evaluate the effect of age, transmission intensity, and nutrition status on the nature of interaction between the two parasites.

## Supplementary Material

Table 1 summarizes key characteristics and findings of studies on *Plasmodium* and helminth co-infection included in this review. For each included study information on the study area, reference number, age group, sample size, study design, helminth species, *Plasmodium* species and main finding about the nature of association of helminth and *Plasmodium* infection is summarized. Majority of the studies were conducted among children in Africa. *P. falciparum* was the most dominant species investigated in majority of the studies.
