# Erratum to “Epidemiology of* Plasmodium* and Helminth Coinfection and Possible Reasons for Heterogeneity”

**DOI:** 10.1155/2017/9281598

**Published:** 2017-02-20

**Authors:** Abraham Degarege, Berhanu Erko

**Affiliations:** Aklilu Lemma Institute of Pathobiology, Addis Ababa University, P.O. Box 1176, Addis Ababa, Ethiopia

In the article titled “Epidemiology of* Plasmodium* and Helminth Coinfection and Possible Reasons for Heterogeneity,” [1] there were errors in the citations of references in Supplementary Table  1, where citation [24] should be replaced with citation [13], and the citations from [26] to [63] should be changed to citations from [25] to [62]. Additionally, the study area in row 38 should be Gabon instead of Nigeria. The correct table is available at the Supplementary Material link.
