# Supplementary material for: Epidemiology of Plasmodium and Helminth Coinfection and Possible Reasons for Heterogeneity
Source: Biomed Res Int. 2016 Mar 22;2016:3083568. doi: 10.1155/2016/3083568 (PMC4820611; doi:10.1155/2016/3083568)
Supplement: Supplementary file 1 — Table 1 summarizes key characteristics and findings of studies on Plasmodium and helminth co-infection included in this review. For each included study information on the study area, reference number, age group, sample size, study design, helminth species, Plasmodium species and main finding about the nature of association of helminth and Plasmodium infection is summarized. Majority of the studies were conducted among children in Africa. P. falciparum was the most dominant species investigated in majority of the studies. [file 3083568.f1.docx]

Table 1: Characteristics of some of the studies included in this review

| Study area  (Reference) | Age group | Sample size | Study design | Helminth  Species | *Plasmodium* Species | Findings |
| --- | --- | --- | --- | --- | --- | --- |
| Comoros  Island [32] | Children | 869 | CS | *Al* | NA | Decreased occurrence of malaria in children heavily infected with ‘*Al*’ compared to those with low ‘*Al*’ burden |
| Comoros  Island [27] | Children | 122 | RCT | *Al* | *Pf* | Increased ‘*Pf*’ incidence and *Plasmodium* density in children treated (51%) than untreated with anthelminthic drugs |
| Senegal [55] | Children | 80 | PC | Pooled STHs | *Pf* | Increased malaria incidence in children infected than those uninfected with STHs (RR= 1.54; p=0.003) |
| Senegal [38] | Children | 210 | CC | *Al* | NA | Increased severe malaria attack in children infected with ‘*Al*’ than those uninfected with ‘*Al*’ (*A*OR= 9.95; 95% CI= 3.03 32.7) |
| Senegal [19] | Children | 512 | PC | *Sm* | *Pf* | Increased ‘*Pf*’ incidence in children infected with ‘*Sm*’ (particularly in those with heavy egg load) compared to those uninfected (RR= 2.24; p=0.01) |
| Senegal [15] | Children | 523 | PC | *Sh*  Pooled STHs | *Pf* | Decreased *Plasmodium* density in children infected with low intensity ‘*Sh*’ compared to those uninfected with helminths (β= -0.34; 95% CI=−0.85,−0.10)  Lack of association between ‘STH’ infection and *Plasmodium* density |
| Mali [6] | Children | 676 | PC | *Sh* | *Pf* | Decreased number of ‘*Pf*’ episodes (1.55) in children infected than uninfected (1.81 infections ) with ‘*Sh*’ in children with ages from 4 to 8 years |
| Madagascar [28] | Children | 350 | RCT | *Al* | *Pf* | Increased ‘*Pf*’’ incidence and density of the parasite in children treated with anthelminthic drugs compared to those not treated. |
| Madagascar [29] | Children | 212 | RCT | *Al* | *Pf* | Increased ‘*Pf’* incidence and density of the parasite (β=0.25; 95% CI=0.07–0.42) in children treated than those not treated with anthelminthic drugs |
| Uganda [43] | Children and adult | 435 | PC | *Al,* Hw, *Tt* | NA | ‘*Al*’, ‘Hw,’ ‘*Tt*’ infections were not associated with the risk of malaria |
| Uganda [46] | Pregnant women | 2507 | CS | Hw, *Sm*, *Tt* | *Pf* | Increased ‘*Pf*’ prevalence in women infected with ‘Hw’ (OR=1.53; 95% CI=1.09, 2.14). However, ‘Sm’ and ‘Tt’ infections were not associated with the occurrence of ‘Pf’ infection. |
| Kenya [63] | Children | 387 | PC | Pooled STHs  *Sh* | *Pf* | STH and ‘*Sh*’ infection were not associated with the risk of ‘*Pf*’ infection |
| Kenya [40] | Pregnant women | 390 | CS | *Al*, Hw, *Tt* | 95.9% *Pf*,  4.1% mixed *Pf*  and *Pv* | ‘*Al*’, ‘Hw’ and ‘*Tt*’ were not associated with the prevalence of *Plasmodium* infection |
| Ethiopia [33] | Children and adult | 458 | CS | *Al*, Hw, *Tt*  Pooled STHs | *Pf*  *Pv* | Intensity of ‘*Al*’ infection was negatively associated with *Plasmodium* density  Intensity of ‘Hw’ infection was positively associated with *Plasmodium* density  ‘*Tt*’ infection didn’t show association with malaria  Prevalence of severe malaria was lower among individuals infected than uninfected with STH (OR=0.317, 95% CI=0.315, 0.86) |
| Ethiopia [17] | Children and adult | 702 | CS | *Al*, Hw, *Tt*  *Sm*  Pooled STH | *Pf*  *Pv* | Increased prevalence of ‘*Pf*’ in individuals infected with ‘*Al*’ compared to those not infected with helminth (AOR= 2.55; 95% CI= 1.40, 4.63)  Increased prevalence of ‘*Pf*’ in individuals infected with ‘*Tt’*  compared to those not infected with helminth ( AOR=2.77: 95% CI=1.15, 6.65)  Increased prevalence of ‘*Pf*’ in individuals infected with ‘*Sm*’ compared to those not infected with helminth (AOR=3.25; 95% CI= 1.32, 7.97)  Increased prevalence of ‘*Pf’*  in individuals infected with any intestinal helminth species ( AOR=2.89; 95% CI=1.77, 4.72)  Prevalence of ‘*Pf*’ didn’t associate with the occurrence of ‘Hw’ infection |
| Zimbabwe  [24] | Children | 1303 | CS | Hw, *Sm*, *Sh*  *Al*, *Tt* | *Pf* | Increased ‘*Pf*’ prevalence in children infected than uninfected with ‘Hw’ (OR = 2.48; 95% CI= 1.56–3.93)  Increased ‘*Pf*’ prevalence in children infected than uninfected with ‘*Sm*’ (OR = 1.85; 95% CI= 1.20–2.87)  Prevalence of ‘Pf was similar between children infected than uninfected with ‘*Tt*’ |
| Gahanna [37] | Pregnant women | 746 | CS | Hw, *Al*, *Tt*  Pooled STHs | *Pf* | Increased ‘*Pf*’ prevalence in women infected with ‘Hw’ (AOR=1.6; 95% CI=1.1, 3.6) and ‘*Al*’ (AOR=3.9; 95% CI=1.5, 7.1) than uninfected  ‘*Tt*’ did not associate with the prevalence of ‘*Pf*’ infection  STH infection was associated with the prevalence ‘*Pf*’ infection (AOR=4.8; 95% CI=3.4, 4.0) |
| Brazil [30] | Children | 216 | PC | *Al*, Hw, *Tt* | *Pv* | The risk of the first clinical malaria was similar between those infected and uninfected with STH  Lack of association between ‘*Tt*’ infection and *Plasmodium* density |
| Thailand [35] | Adults | 537 | CC | *Al* | *Pf* | Low cerebral malaria, renal failure and pulmonary edema in patients infected than uninfected with ‘*Al*’ (AOR= 0.25; 95% CI=0.009, 0.67) |
| Thailand [57] | Adults | 177 | CC | Pooled STHs | NA | The odds of malaria related renal failure (AOR= 0.16, 95% CI=0.03, 0.85) and jaundice (AOR=0.39, 95% CI=0.16, 0.96) was less in patients infected than uninfected with helminths |
| Thailand [58] | Adults | 98 | CC | Pooled STHs | NA | Low prevalence of cerebral malaria in patients infected than those uninfected with helminths (AOR= 0.36; 95% CI = 0.19, 0.7) |
| Thailand [36] | Adults | 928 | CS | *Al* | *Pf* and *Pv* | Higher prevalence of mixed ‘*Pf*’ and ‘*Pv*’ infection in patients infected than uninfected with ‘*Al*’ (AOR=3.6; 95% CI=1.2, 11.1) |
| Thailand [54] | Adults | 731 | PC | Pooled STHs | *Pf* and *Pv* | Increased incidence of ‘*Pf*’ malaria in patients infected than uninfected with helminths (ARR=2.24; 95% CI=1.4, 3.6) |
| Thailand [60] | Adults | 307 | CS | Pooled STHs | *Pf* | Increased gametocyte carriage in patents infected than uninfected with STH (AOR=2; 95% CI=1.03–3.8) |
| Thailand [34] | Adults | 119 | CS | *Al* | *Pv* | ‘*Al*’ infection was inversely related with patients fever |
| Thailand [31] | Pregnant women | 829 | CS | Hw, *Al* | *Pf* and *Pv* | Increased risk of malaria in women infected with ‘Hw’(AOR=1.66; 95% CI= 1.06, 2.60)  Decrease risk of malaria in women infected with ‘*Al*’ (AOR=0.43; 95% CI=0.23, 0.84) |
| Thailand [50] | Adults | 248 | CS | *Al*, Hw, *Tt* | *Pf* | Increased multiplicity of ‘*Pf’* infection in patients infected than uninfected with ‘*Tt*’ (AOR=2.45; 95 CI=1.14–5.22)  The number of clones of ‘*Pf*’ was similar between patients infected and uninfected with ‘Hw’  The number of clones of ‘*Pf*’ was similar between patients infected and uninfected with ‘*Al*’ |
| Tanzania [52] | Children | 1033 | CS | Pooled STH | *Pf* and *Pv* | ‘STH’ infection was positively associated with the prevalence of *Plasmodium* infection (AOR=1.4, 95% CI =1.0-2.1) |
| Tanzania[49] | children | 1,546 | CS | Hw | *Pf* | Prevalence of ‘*Pf*’ infection was similar between children who were infected and uninfected with ‘Hw’ |
| Ethiopia, Uganda and Kenya [47] | Children | 28050 | CS | Hw, *Sm*, *Sh* | *Pf* and *Pv* | ‘Hw’ infection was associated with an increased prevalence of ‘*Pf*’ infection  There was no association between *Schistosoma* and ‘*Pf*’ infection |
| Colombia  [42] | Children and adult | 246 | CC | *Al*, Hw, *Tt* | *Pf* | Hw infection was associated with an increased prevalence of ‘*Pf*’ infection (AOR=4.36; 95% CI= 1.68–11.31)  There was no association between ‘*Al’* and ‘*Pf*’’ infection  There was no association between ‘*Tt’* and ‘*Pf*’ infection |
| Nigeria [59] | Children and adult | 1442 | CC | *Al*, Hw, *Tt* | *Pf* | Helminth infection was associated with a decreased prevalence of malaria  (AOR=0.50; 95% CI= 0.2–0.84 |
| Senegal [26] | Children | 178 | PC | *Sm* | *Pf* | ‘*Pf*’ density was similar between children with moderate or heavy intensity ‘*Sm*’ and those who were not infected with ‘*Sm*’.  ‘*Pf*’ density was lower in children with light intensity ‘*Sm*’ compared to children who were not infected with ‘*Sm*’(β=-0.28; 95% CI=-0.52, -0.04) |
| Nigeria [41] | Children | 690 | RCT | *Al* | *Pf* | Prevalence and density of *‘Pf’* was similar between children infected and uninfected with ‘*Al*’ |
| Côte d'Ivoire  [48] | Children adult | 324 | CS | Hw | *Pf* | Prevalence of ‘*Pf*’ was higher in children of age 6-8 years who were infected  than uninfected with ‘Hw’ (AOR=7.47; 95% CI=1.84, 30.32) |
| Cameroon [62] | Children | 263 | CS | *Al*  *Tt* | *Pf* | Prevalence of ‘*Pf*’ was similar between children infected and not infected with STH |
| Senegal [53] | Children | 203 | PC | *Hw*, *Al*  *Tt* | *Pf* | Increased odds of ‘*Pf*’ infection in children infected than uninfected with STH (AOR=2.69, 95% CI=1.34, 5.39) |
| Nigeria [14] | Children | 125 | CS | *Sh* | *Pf* | Prevalence of ‘*Pf*’ was similar between children infected and uninfected with ‘*Sh*’ |
| Senegal [16] | Children | 234 | PC | *Sh* | *Pf* | The risk of ‘*Pf*’ infection and density of the parasite was similar between children infected and uninfected with ‘*Sh*’ |
| Ethiopia [8] | Children | 387 | CS | *Sh* | *Pf* | Prevalence of ‘*Pf*’ infection was higher among children infected than uninfected with ‘*Sh*’ (OR:=2.8; 95% CI= 1.21, 6.5) |
| Mali [9] | Children | 616 | PC | *Sh* | *Pf* | Prevalence of ‘*Pf*’ infection was higher among children infected than uninfected with ‘*Sh*’ (OR=3.23, 95% CI= 1.76, 5.90) |
| Kenya [10] | Children | 223 | CS | *Sh* | *Pf* | Prevalence of ‘*Pf*’ infection was higher among children infected than uninfected with ‘*Sh*’ (OR=1.67,95% CI=1.46, 1.99) |
| Uganda [18] | Children | 5000 | CS | *Sm* | *Pf* | Prevalence of ‘*Pf*’ infection was higher among children infected than uninfected with ‘*Sm*’ (OR: 2.16, 95% CI: 1.89, 2.47). |

Al: *Ascaris lumbricoides*; CC: Case Control; CS: Cross-sectional; Hw: hookworm; NA: not available/not provided/not mentioned/not speciﬁed; OR: odds ratio; Pf: *Plasmodium falciparum*; Pv: *Plasmodium vivax*; RCT: randomized control trial; RR: Relative risk; Sh: *Schistosoma haematobium*; Sm: *Schistosoma manosni*; STH: soil-transmitted helminths; Tt: *Trichuris trichiuria*; PC: Prospective control
